# Cerebellar Contributions to Spatial Learning and Memory: Effects of Discrete Immunotoxic Lesions

**DOI:** 10.3390/ijms26199553

**Published:** 2025-09-30

**Authors:** Martina Harley Leanza, Elisa Storelli, David D’Arco, Gioacchino de Leo, Giulio Kleiner, Luciano Arancio, Giuseppe Capodieci, Rosario Gulino, Antonio Bava, Giampiero Leanza

**Affiliations:** 1 Neurogenesis and Repair Lab., Department of Drug and Health Sciences, University of Catania, Via S. Sofia 64, 95125 Catania, Italyluciano.arancio@hotmail.it (L.A.);; 2 Neurogenesis and Repair Lab., Basic Research And Integrative Neuroscience (B.R.A.I.N.) Centre, Department of Life Sciences, University of Trieste, Via Fleming 2, 34127 Trieste, Italy; 3 Neurophysiology Lab., Department of Biomedical and Biotechnological Sciences, University of Catania, Via S. Sofia 89, 95123 Catania, Italy; 4 Molecular Preclinical and Translational Imaging Research Centre—IMPRonTE, University of Catania, 95123 Catania, Italy

**Keywords:** acetylcholine, basal forebrain, cerebellum, Purkinje cells, immunotoxin, reference and working memory, Alzheimer’s disease, rat

## Abstract

Evidence of possible cerebellar involvement in spatial processing, place learning and other types of higher order functions comes mainly from clinical observations, as well as from mutant mice and lesion studies. The latter, in particular, have reported deficits in spatial learning and memory following surgical or neurotoxic cerebellar ablation. However, the low specificity of such manipulations has often made it difficult to precisely dissect the cognitive components of the observed behaviors. Likewise, due to conflicting data coming from lesion studies, it has not been possible so far to conclusively address whether a cerebellar dysfunction is sufficient per se to induce learning deficits, or whether concurrent damage to other regulatory structure(s) is necessary to significantly interfere with cognitive processing. In the present study, the immunotoxin 192 IgG-saporin, selectively targeting cholinergic neurons in the basal forebrain and a subpopulation of cerebellar Purkinje cells, was administered to adult rats bilaterally into the basal forebrain nuclei, the cerebellar cortices or both areas combined. Additional animals underwent injections of the toxin into the lateral ventricles. Starting from two–three weeks post-lesion, the animals were tested on paradigms of motor ability as well as spatial learning and memory and then sacrificed for post-mortem morphological analyses. All lesioned rats showed no signs of ataxia and no motor deficits that could impair their performance in the water maze task. The rats with discrete cerebellar lesions exhibited fairly normal performance and did not differ from controls in any aspect of the task. By contrast, animals with double lesions, as well as those with 192 IgG-saporin given intraventricularly did manifest severe impairments in both reference and working memory. Histo- and immunohistochemical analyses confirmed the effects of the toxin conjugate on target neurons and fairly similar patterns of Purkinje cell loss in the animals with cerebellar lesion only, basal forebrain-cerebellar double lesions and bilateral intraventricular injections of the toxin. No such loss was by contrast seen in the basal forebrain-lesioned animals, whose Purkinje cells were largely spared and exhibited a normal distribution pattern. The results suggest important functional interactions between the ascending regulatory inputs from the cerebellum and those arising in the basal forebrain nuclei that would act together to modulate the complex sensory–motor and cognitive processes required to control whole body movement in space.

## 1. Introduction

The cerebellum has classically been referred to as a structure involved entirely in modulating the planning, execution and co-ordination of motor activity for skilled voluntary movements, as well as posture, muscle tone and gait [1,2]. In recent decades, however, this view has been challenged by evidence indicating that this structure might also be involved in attention, learning, language and cognition [3,4,5]. The hypothesis that the cerebellum could be involved in high-order processes is supported by two major findings: (i) the presence of neuropsychological deficits in patients with discrete cerebellar pathology and (ii) the cerebellar activation in cognitive, attentional and language tasks, as assessed by brain imaging [4,6,7,8,9]. Within such a framework, Purkinje cells (which provide the main output of the cerebellum) appear to play a major role in that they may direct information to other areas that are critical for the acquisition and storage of spatial information such as the limbic system, superior colliculus, parietal cortex and the dorsolateral prefrontal cortex. Notably, a loss of Purkinje cells would disrupt the output of the cerebellum to those areas [10,11], possibly contributing to cerebellar atrophy reported in the advanced-late stages of Alzheimer’s Disease (AD [12,13,14,15,16]).

The issue of cerebellar involvement in spatial processing has been addressed using a variety of animal models and well-established paradigms of spatial learning and memory. Among these, mutant mice strains with different types of cerebellar degeneration exhibit specific patterns of Purkinje cell loss as well as deficits of varying severity in tasks involving spatial navigation and spontaneous alternation [17,18]. However, on anatomical grounds, a clear association between cerebellar abnormalities and cognitive dysfunction has often been problematic in these animals, which also exhibited potential developmental issues. In 1998, Petrosini and colleagues reported severe navigation deficits in hemicerebellectomized rats. They suggested the role of the cerebellum as a part of a larger system including the frontal cortex, posterior parietal cortex, inferior temporal cortex, hippocampus and basal ganglia and demonstrated how cerebellar lesions in rats appeared to induce deficits in the procedural components of the spatial function, having the declarative ones still intact [19]. While the conclusions of this study have certainly been of great value to address the involvement of distinct cerebellar regions in the regulation of complex behavioral manifestations, the lesioning technique is quite invasive and the arising functional deficits have not always proven easy to frame within a cognitive domain. In fact, in a subsequent report, the same authors stated that cerebellar networks appear indispensable for acquiring complex behaviors, implying that extensive cerebellar lesions may interfere with animals’ ability to create a representation of a novel environment, as the lesion makes them unable to explore it properly [20]. Thus, it has become increasingly clear that investigating the cerebellar contribution to cognitive functions in experimental animals would necessarily require the use of tools able to produce more specific lesions without disrupting normal motor behavior. In such a framework, a more specific and discrete Purkinje cell loss has been obtained by using an immunolesioning approach, in which a ribosome-inactivating protein (saporin, extracted from *Saponaria officinalis*) is coupled to a monoclonal antibody directed to an antigen expressed onto cerebellar Purkinje cells. In initial studies, the immunotoxin OX7-saporin was used, where the saporin is conjugated with the monoclonal antibody OX7 that recognizes Thy-1.1, a neuronal surface glycoprotein. When given intraventricularly, the toxin produced a selective destruction of cerebellar Purkinje cells that resulted in rather mild acquisition deficits in a standard Morris water maze task [21], but did not affect working memory performance in a radial arm maze task [22]. Notably, a robust Purkinje cell loss has also represented a major confounding factor in functional studies, using the selective cholinergic immunotoxin 192 IgG-saporin to model in experimental animals the marked cortical/hippocampal cholinergic denervation seen in AD as a result of cholinergic neuronal degeneration in the basal forebrain. The toxin is a conjugate of saporin with a monoclonal antibody targeting the low affinity Nerve Growth Factor (NGF) receptor (p75NTR), specifically expressed onto the surface of basal forebrain cholinergic neurons, but also on selected subpopulations of Purkinje cells in the adult rat [23,24]. Thus, after intraventricular infusion, the toxin causes the concomitant destruction of p75NTR-expressing Purkinje cells, in addition to inducing cholinergic depletion [25,26,27]. Conversely, no such cerebellar damage and, more importantly, no obvious cognitive impairment is detectable when the 192 IgG-saporin is infused directly into the basal forebrain nuclei [28,29,30,31,32] or given intraventricularly to developing animals [33,34,35], in spite of comparable cholinergic neuronal and terminal fiber loss. In 1999, Waite and coworkers sought to dissociate the cognitive/attentional effects related to Purkinje cell loss from those due to cholinergic depletion using the 192 IgG-saporin and the OX7-saporin to, respectively, lesion the basal forebrain cholinergic neurons and the cerebellar Purkinje cells. These authors observed differential effects of either lesion, but also inconsistent behavioral profiles in animals with cerebellar loss only as opposed to animals displaying 192 IgG-saporin-induced BF and cerebellar loss, most probably due to the fact that the OX7- saporin and the 192 IgG-saporin may target different populations of cerebellar Purkinje cells [36]. With the purpose of overcoming such limitations, the aim of the present study was to analyze the behavioral and anatomical effects of discrete injections, in different groups of animals, of the same 192 IgG-saporin toxin into the basal forebrain nuclei and/or into the cerebellar vermis and hemispheres. By carefully choosing dose, injection sites and highly sensitive testing paradigms, we thus sought to better mirror, both in terms of anatomical pattern and functional outcome, the cholinergic and Purkinje cell depletion normally seen following intraventricular administration of the toxin conjugate, so as to possibly dissect out their distinct or concurrent contribution to spatial learning and memory.

## 2. Results

### 2.1. General Observations

Consistently, the rats with vehicle injections did not differ from the intact animals on any of the behavioral or morphological parameters analyzed. These animals were therefore combined into a single Control group (n = 12) for statistical analyses and illustrations. The other groups comprised animals with the following: (i) bilateral intraventricular injection of 192 IgG-saporin (ICV, n = 12); (ii) bilateral injections of 192 IgG-saporin into the basal forebrain nuclei (BF, n = 12); (iii) injections of 192 IgG-saporin into the cerebellar hemispheres and vermis (CBL, n = 12); and (iv) injections of 192 IgG-saporin into both the basal forebrain nuclei and cerebellar hemispheres and vermis (BF/CBL, n = 12).

All animals, irrespective of their treatment, exhibited a steady increase in body weight and fairly normal sensory–motor performance in both bridge and grid tests (Table 1). Thus, no deficits in locomotion or motor coordination were detected that would affect performance in the water maze task.

### 2.2. Effects of the Lesions on Spatial Navigation

#### 2.2.1. Effects on Cued Navigation and Reference Memory

**Figure 1:**
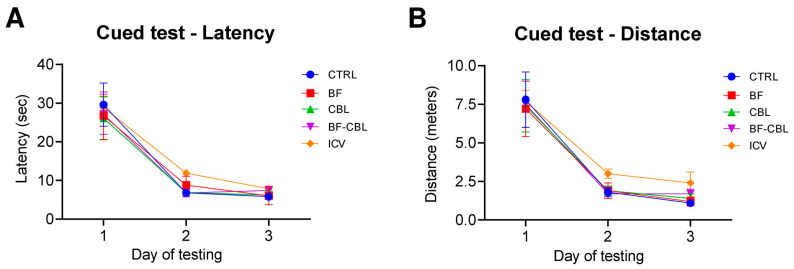
The immunotoxin lesion does not cause sensory (e.g., visual) deficits. Escape latency (**A**) and swim distance (**B**) recorded in the cued version of the MWM. Each point represents the mean value ± standard error of the mean (SEM) for the four trials administered on each of the three days of testing.

Figure 1A,B illustrates the performance in the cued version of the Morris water maze task, when the platform location was rendered visible and the animals could swim towards it by using the visual cues available from within the pool. The animals in all groups significantly reduced the time and distance required to locate the platform over the three testing days (two-way mixed ANOVA, effect of day for latency, F2,110 = 62.7; for distance, F2,110 = 65.7; both *p* < 0.001) and did not differ from each other (main group effect, for latency: F4,55 = 0.5; for distance: F4,55 = 0.4; *p* < 0.001; group x day, for latency, F8,110 = 0.1; for distance, F8,110 = 0.2, all n.s.). Thus, most likely, no non-cognitive (e.g., visual) impairments were produced by the lesions that would have any overall impact on the navigation ability of the animals.

**Figure 2:**
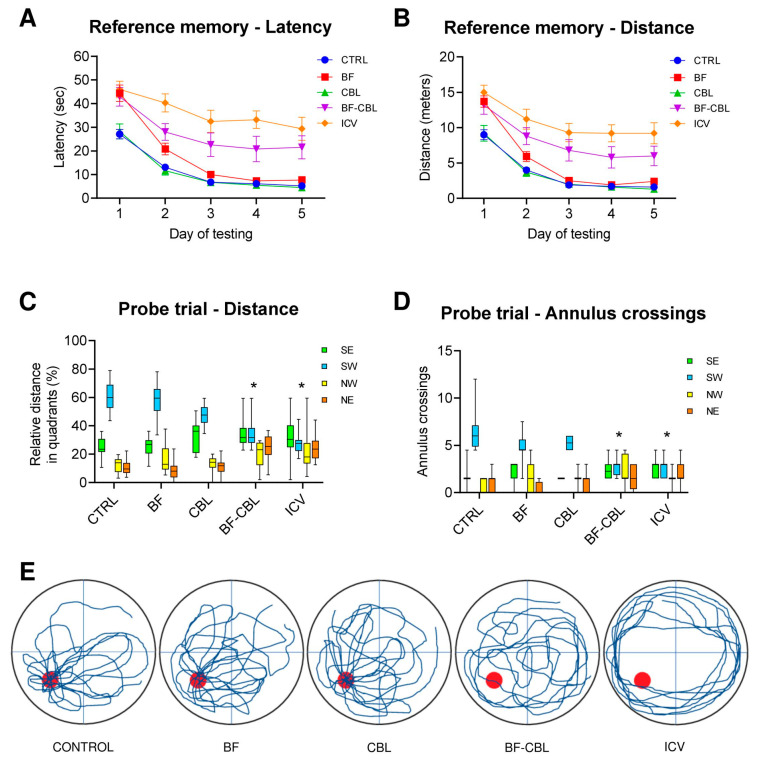
Morris water maze, reference memory test at about 4–5 weeks post lesion. Average escape latencies (**A**) and swim distances (**B**) required the animals in the various groups (n = 12 in each group) to locate the submerged platform during the acquisition of the spatial navigation task. Each point represents the mean value for the block of four trials on each of the five consecutive days of testing (±SEM). Lower diagrams illustrate the mean relative distance swum (**C**) and the average number of annulus crossings (**D**) in each quadrant during the spatial probe trial, upon removal of the escape platform. In (**E**), the actual swim paths taken by representative rats from the different groups are illustrated. Similarly to Control, animals in the BF or CBL lesion groups exhibited equally efficient performances and a pronounced bias for the original platform site (red dot) in the training (SW) quadrant. By contrast, the BF-CBL double-lesioned and the ICV animals appeared severely impaired. Asterisks indicate significant difference from the Control group at *p* < 0.05.

Animals’ performance during the place test, when the platform was below the water surface and the animals had to use extra maze cues to locate it, is shown in Figure 2A,B. Initially all animals required about 27–46 s and 9–15 m to locate the hidden platform but improved significantly with daily training (two-way mixed ANOVA, effect of day for latency, F4,220 = 124.5; for distance, F4,220 = 156.6; both *p* < 0.001), reaching an asymptotic performance over the last 3 days of testing. Notably, the animals in the Control, BF and CBL groups learned to locate the hidden platform more rapidly with training and were able to swim directly to the platform in about 6–8 s and 2–2.5 m. By contrast, the rats in the BF-CBL and the ICV groups performed less efficiently and took significantly longer time and distance to locate the platform (main group effect, for latency, F4,55 = 18.8; for distance, F4,55 = 17.1; both *p* < 0.001; group x day, for latency, F16,220 = 3.2; *p* < 0.001; for distance, F16,220 = 2.4, *p* < 0.002), particularly during the last four testing days (Tukey post hoc test *p* < 0.05 for both latency and distance). Swim speed, recorded as a screen of motor ability during the execution of the navigation task, did not differ between groups (one-way ANOVA, *p* > 0.1 n.s.) and averaged about 0.3 m/s across all testing days. Figure 2C,D illustrates animals’ ability to locate the platform site on the final spatial probe trial on day 5, when the platform was removed and the animals were allowed to swim freely for 60 s. In general, the animals in the Control, BF and CBL groups swam primarily in the training (SW) quadrant and did not differ from each other, whereas those in the BF-CBL and ICV groups showed no such spatially focused search behavior and tended to distribute their swimming equally in all quadrants. Two-way mixed ANOVA on the distance and annulus crossings measures revealed a significant effect of quadrant (for distance, F3,165 = 92.6; for annulus crossings, F3,165 = 74.8; both *p* < 0.001), as well as a group–quadrant interaction (for distance, F12,165 = 12.6; for annulus crossings, F12,165 = 9.1; both *p* < 0.001). Subsequent pairwise comparisons (Tukey post hoc test) confirmed the lack of any clear place preference among the animals in the BF-CBL and ICV groups, whose swimming was either thigmotaxic or outside the training quadrant (*p* < 0.05 vs. Control). Interestingly, no difference between groups was observed in the total number of annulus crossings in all quadrants (F4,55 = 1.2; *p* > 0.3, n.s.), indicating an equally active, albeit not focused, swimming behavior in all animals (see also the actual swim paths from representative animals in Figure 2E).

#### 2.2.2. Effects on Working Memory

**Figure 3:**
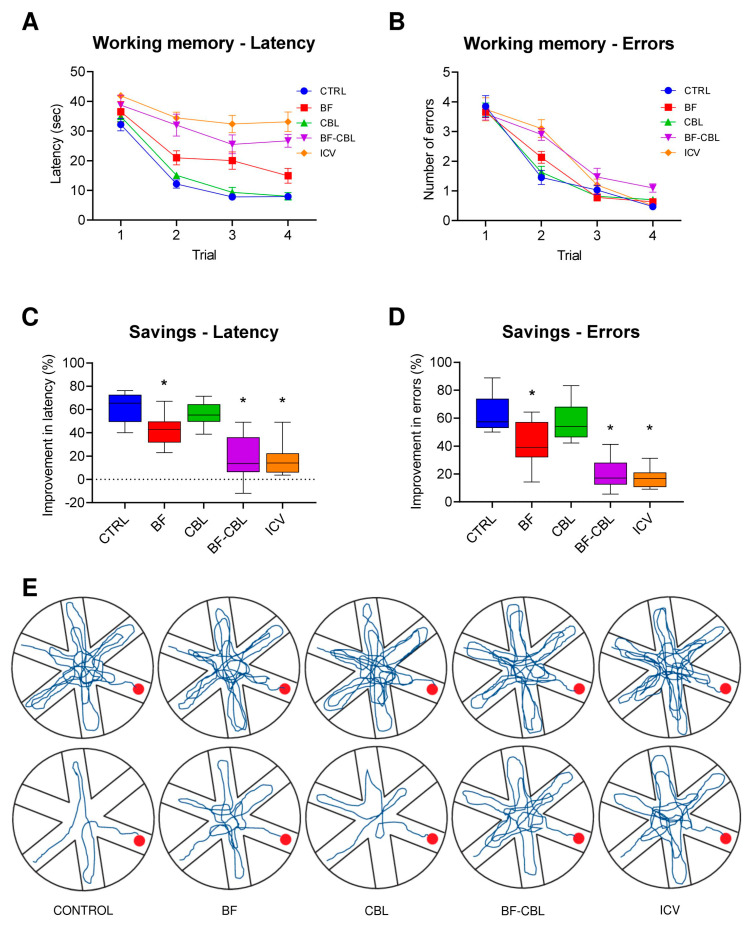
Radial arm water maze, working memory performance at about 5–6 weeks post-lesioning. Latency (**A**) and number of entry errors (**B**) required by the five groups of animals (n = 12 in each group) to find the hidden platform in the radial arm water maze task. Each sample point represents the mean latency and errors recorded during each 60 s trial over four consecutive testing days (±SEM). In the lower diagrams, performances are plotted as percentage improvement (savings) between trials 1 and 2 for latency (**C**) and errors (**D**). In (**E**), the actual swim paths taken by representative animals from the different groups to find the platform (red dot) are illustrated. Note the marked impairments in the BF-CBL and ICV groups. Asterisks indicate significant difference from Control group at *p* < 0.01.

Animals’ performance in the radial arm water maze, i.e., the working memory version of the spatial navigation task, is illustrated in Figure 3A,B. In this test, the position of the platform is randomly moved to a different arm on each testing day, prompting the animals to adopt a novel search strategy every day in order to re-learn its position. In general, there was a reduction in the overall latency and entry errors over the four trials (two-way mixed ANOVA, effect of trial on latency, F3,165 = 178.8; on errors F3,165 = 276.0; in both cases *p* < 0.001), but with a varying pattern of efficiency (group x trial interaction for latency F12,165 = 6.8; for errors, F12,165 = 4.2; in both cases *p* < 0.001), indicating differences among groups for their ability to perform in this task. Close inspection of the acquisition curve indicated that rats in the Control and CBL groups reduced rapidly the latency and the entry errors made across trials (particularly between trials 1 and 2) to locate the platform, thus displaying intact working memory abilities. The animals in the BF groups appeared somewhat less efficient in relocating the platform, whereas the rats in the BF-CBL and ICV group did not show any obvious improvements, their performance being clearly impaired (main group effect for latency, F4,55 = 18.9; *p* < 0.001; for errors, F4,55 = 3.2; *p* < 0.05; post hoc comparisons with the Control and CBL groups, *p* < 0.05).

Saving measures for latency and entry errors (calculated as percentage improvement between the first and the second trial across days) were used to provide an additional screen of performance efficiency in the RAWM task. As shown in Figure 3C,D, these analyses revealed that the Control and CBL animals reduced their latency and arm selection errors by about 56–62% and 57–63%, respectively, and did not differ from each other. The animals in the BF group exhibited a relatively—but significantly—lower 42% improvement compared to those in the Control and CBL groups. Notably, the percent improvements measured in the BF-CBL and ICV groups were seen as dramatically lower (one-way ANOVA, *p* < 0.001 followed by Tukey post hoc test at *p* < 0.05 for both measures) and never exceeded 17–19% (see also the actual swim paths from representative animals in Figure 3E).

### 2.3. Morphological Analyses

#### 2.3.1. Effects on ChAT-Immunoreactive Neurons in the Basal Forebrain Nuclei

**Figure 4:**
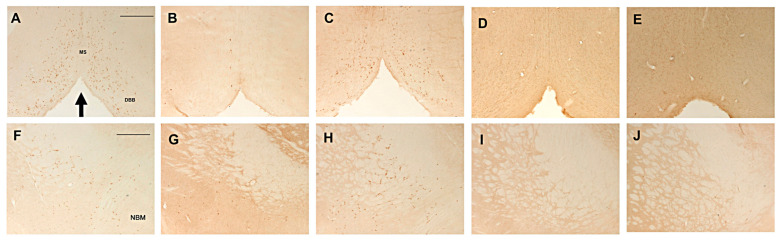
Representative examples of choline acetyltransferase (ChAT) immunostaining in coronal sections from the septum/vDBB (**A**–**E**) and NBM (**F**–**J**) in the various treatment groups. At about 8–10 weeks post-lesioning, marked depletions of ChAT immunoreactive neurons were evident in the animals from the BF (**B**,**G**), the BF-CBL (**D**,**I**) and the ICV groups (**E**,**J**), whereas the same regions appeared unaffected in the CBL animals (**C**,**H**), compared to Controls (**A**,**F**). The arrow in (**A**) indicates the midline. Scale bars in (**A)** and (**F**) (for **A**–**J**): 500 μm.

In all animals receiving the bilateral administration of 192 IgG-saporin locally into the basal forebrain nuclei, or into the lateral ventricles, only sparse ChAT-immunoreactive (i.e., cholinergic) neurons could be scattered into both the sept/vDBB and NBM, compared to Control [26,37,38]. By contrast, no clear-cut neuronal loss could be detected following injections of the immunotoxin into the cerebellar vermis and cortices alone (Figure 4A–J). Stereological analyses revealed a dramatic 94–98% loss of ChAT immunoreactive neurons in the animals of the ICV, BF and BF-CBL groups, compared to Control or CBL-injected animals (Table 2), whereas the parvalbumin-immunoreactive (i.e., GABAergic) neuronal population in the septum/DBB was unaffected (e.g., [26,29]).

#### 2.3.2. Effects on AChE-Positive Innervation in Neocortex and Hippocampus

**Figure 5:**
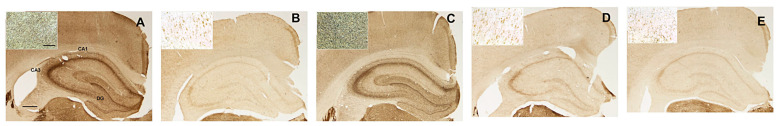
Representative examples of acetylcholinesterase (AChE) histochemistry showing, on the coronal plane, the extent of cholinergic denervation, in the various treatment groups, at about 8–10 weeks post-lesioning. Note the dramatic denervation induced by the toxin-conjugate in the neocortex and hippocampus of the animals in the BF (**B**), the BF-CBL (**D**) and the ICV groups (**E**) compared to the normal pattern exhibited by the Control (**A**) and the CBL groups (**C**). Cx, fronto-parietal cortex; CA, cornu ammonis of the hippocampus; CC, corpus callosum; DG, dentate gyrus. Scale bar in (**A)** (for **A**–**E**): 500 μm; scale bar in the insert in (**A**) (for all the inserts): 50 μm.

Consistent with previous observations (e.g., [25,26,36,37]), bilateral intraventricular administration of 192 IgG-saporin resulted in a dramatic loss of AChE-positive staining throughout the hippocampus, as well as the frontal and parietal cortices which averaged 70–80%, as estimated by densitometry. A very similar pattern of widespread cholinergic depletion in neocortical and hippocampal areas was exhibited also by the animals in the BF and the BF-CBL groups, receiving the toxin conjugate into the MS/vDBB and NBM nuclei or, combinedly, into these nuclei and the cerebellar vermis and cortices. Notably, injection of the 192 IgG-saporin immunotoxin only into the cerebellar vermis and cortices produced no detectable loss of AChE-positive fibers in any cortical or hippocampal region, their pattern being indistinguishable from Control (Figure 5A–E and Table 2).

#### 2.3.3. Effects on Calbindin-Positive Cerebellar Neurons

**Figure 6:**
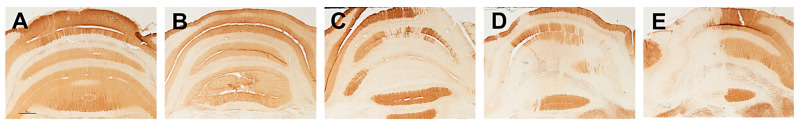
Representative examples of calbindin immunostaining in coronal sections of the cerebellar vermis from animals in the various treatment groups. Note the dramatic depletion of p75NTR-bearing, calbindin-positive Purkinje cells, with a fairly similar band-like pattern, in animals from the CBL (**C**), the BF-CBL (**D**) and the ICV groups (**E**), compared to Control (**A**) and the BF groups (**B**). Scale bar in (**A**) (for **A**–**E**): 500 μm.

Animals in the CBL, BF-CBL and ICV groups injected with the 192 IgG-saporin immunotoxin exhibited a marked loss of calbindin-immunoreactive Purkinje cells, whose pattern appeared fairly similar, irrespective of the administration site or route, and was seen as particularly prominent in the cerebellar lobules 5 and 6, where it assumed a band-like appearance (Figure 6A–E). As estimated by stereology, the loss of Calbindin-immunoreactive neurons in the cerebellar vermis and hemispheres of animals in the ICV, CBL and BF-CBL groups averaged about 61–66%, being relatively mild but significant, compared to Control or BF-injected animals (Table 2).

## 3. Discussion

The 192 IgG-saporin immunotoxin, introduced by Ronald Wiley and his coworkers in the early 1990s [39], has represented a major methodological advancement for studies aimed at modeling AD-like cholinergic loss in experimental animals within the framework of the so-called cholinergic hypothesis [40]. However, interpretation of the toxin-induced cognitive deficits has been confounded by the observation that damage to subpopulations of cerebellar Purkinje cells, namely those expressing p75NTR onto their surface, is practically unavoidable, when the toxin is given intraventricularly to adult animals [25,26,27,28]. By contrast, when ablation of basal forebrain cholinergic neurons was carried out with intraparenchymal injections, ensuring a more precise anatomical definition [28,29,30,31,32,41], or when the toxin was given icv to developing animals [33,34,35], both procedures consistently sparing cerebellar Purkinje cells, only modest or no deficits were detected, raising interesting questions about the possible role played by these cells in cognitive processing.

In the present study, we used a careful and highly selective lesioning approach in conjunction with well-established swim maze tasks to investigate the contribution of distinct subpopulations of cerebellar Purkinje cells, i.e., those expressing the p75NTR, to spatial learning and memory functions, assuming that other adjacent Purkinje cells, apparently with different neurochemical properties and thus not targeted by the toxin, may not be involved in cognition. We hypothesized that a functional interaction between subpopulations of cerebellar Purkinje cells and cholinergic neurons in the MS/vDBB and NBM may be critical for maintaining normal memory performance and thus that their concurrent ablation (e.g., similar to that seen following intraventricular infusion of the 192 IgG-saporin immunotoxin) might represent a necessary condition to yield more severe cognitive impairments than those obtained following ablation of either population alone.

We found here that lesioning the p75NTR-expressing subpopulation of cerebellar Purkinje cells or the cholinergic neurons in the BF by direct intraparenchymal injections of the 192 IgG-saporin toxin into either target did not affect the animals’ ability to perform in the reference memory version of the Morris water maze, where the spatial relationship between the platform location and the external cues remain constant throughout the testing period. Notably, however, marked deficits in both acquisition and retention of the reference memory task were exhibited by the animals with combined toxin injections within the basal forebrain nuclei and cerebellar cortices, whose severity was indistinguishable from that typically seen following intraventricular administration.

In the radial arm water maze task, where the platform position is changed daily and the animals must temporarily retain information on the already visited arms, therefore imposing higher demands on task execution, the animals with cerebellar lesion alone did not differ from Control, whereas those with BF lesions showed a mild deficit. Again, the double-lesioned animals and those with intraventricular injections of the toxin were markedly impaired and did not differ from each other.

The observation of mild, albeit significant, working memory deficits in the animals of the BF group is in keeping with previous findings [42,43] and suggests that damage to both the septo-hippocampal and basalo-cortical components of the basal forebrain cholinergic projection system may be required to obtain some measurable effects on specific aspects of cognitive function, namely attention and working memory.

Notably, the more severe deficits seen here upon lesioning specific subpopulations of cerebellar Purkinje cells (either by local injections or by diffusion following intraventricular administration) point at this cerebellar neuronal population as actively interacting with the cholinergic basal forebrain projection systems in the regulatory control of aspects of learning and memory function. This is of importance, in light of recent functional neuroimaging data confirming early tract-tracing studies [44,45] about the existence of functional connectivity networks linking cortical regions and distinct cerebellar zones [46,47,48]. Likewise, a striking feature of the present cerebellar 192 IgG-saporin lesion model (both when the toxin is given icv or locally infused) is that Purkinje cell loss does not occur at random but rather in an ordered array of bands, with Purkinje cells of specific molecular phenotypes dying (about 50–60%) and other surviving, which is consistent with the idea that distinct neuronal populations (about one-half) of the cerebellum may be involved in cognitive control [47]. In such framework, cerebellar changes, as those detected here following treatment with the 192 IgG-saporin immunotoxin, may be more pervasive than previously thought, with respect to other regulatory dysfunctions, and thus critical for the understanding of the clinical pathological correlates in a neurodegenerative condition such as AD [49].

## 4. Materials and Methods

### 4.1. Animals and Experimental Design

A total of sixty young adult female Sprague-Dawley rats, provided by the animal facility at the University of Trieste, were used. The gender assures a more steady increase in body weight compared to males and better reliability during handling. The animals weighed 225–250 g at the beginning of the experiments and were housed in high-efficiency, particulate air-filtered cage units (Tecniplast, Buguggiate, Italy) under standard conditions of light, temperature and humidity, with ad libitum access to food and water. All the experimental procedures were approved by the Ethical Committee at the University of Trieste and closely followed the Italian Guidelines for Animal Care (D.L. 116/92 and 26/2014), and the European Communities Council Directives (2010/63/EU).

The animals were randomly subjected to either of the following treatments: (i) bilateral intraventricular injection of 192 IgG-saporin (ICV, n = 12); (ii) bilateral injections of 192 IgG-saporin into the basal forebrain nuclei MS/vDBB and NBM (BF, n = 12); (iii) injections of 192 IgG-saporin into the cerebellar hemispheres and vermis (CBL, n = 12); (iv) injections of 192 IgG-saporin into both the basal forebrain nuclei and cerebellar hemispheres and vermis (BF/CBL, n = 12). A group of sham-lesioned animals received bilateral injections of vehicle solution into the basal forebrain and cerebellum (Vehicle, n = 6) and other animals were not injected, serving as unoperated (Intact, n = 6) controls.

Starting from about two weeks post-surgery and for the subsequent two–three weeks, the animals underwent weekly assessments of limb strength and coordination, prior to being evaluated for both reference and working memory abilities in the Morris water maze task. After completion of the last testing sessions, at about 8 weeks post-surgery, the animals under terminal anesthesia were perfused and the brains processed for quantitative histo- and immunohistochemistry.

### 4.2. Lesion Surgery

All surgical procedures were carried out on deeply anesthetized animals (sodium pentobarbital, 40 mg/kg i.p.). Intraventricular administration of the toxin was performed as described previously [26,37]. Briefly, 5 µg of 192 IgG-saporin (Advanced Targeting System, Carlsbad, CA, USA) were dissolved in 7 + 7 µL of sterile Hank’s balanced salt solution (HBSS) and injected into the lateral ventricles at the following coordinates (in mm, according to [50]): AP = −0.6 (from bregma); L = ±1.5 (from the midline); V = 4.5 (from the skull surface), with the tooth bar set at 3.3 below the level of the interaural line. The toxin conjugate was injected at a speed of 1 µL/second using a 10 µL Hamilton microsyringe (Hamilton, Bonaduz, Switzerland) and allowing 1 min for diffusion before the cannula was retracted.

Intracerebral administration of 192 IgG-saporin in the MS/vDBB and NBM was carried out as described previously [38]. For the MS/vDBB lesion, 0.5 µL of the immunotoxin (0.160 µg/µL HBSS) were infused per side at the following coordinates: AP = +0.5; L = ±0.6; subdivided into two deposits, V = −7.7 (0.3 µL) and V = −6.1 (0.2 µL); tooth bar = −3.3. For the NBM lesion, 0.7 µL of the toxin (0.142 µg/µL) were infused per side at AP = +1.0; L = ±3.2; V = −7.5 (tooth bar + 5.0). In order to model the partial Purkinje cell loss which occurs following icv injections of the immunotoxin [25,37], discrete infusions of 192 IgG-saporin (0.08 µg/0.5 µL) were carried out at three different sites into the cerebellar vermis and both hemispheres at the following coordinates: AP = −10.8; L = −0.5; ±4; V = −1.4; tooth bar: −3.3. In pilot experiments, the dose and placement sites adopted produced a pattern of Purkinje cell loss consistently equivalent to that observed following intraventricular administration of the immunotoxin. Each parenchymal infusion was carried out over 3 min, waiting for an additional 2 min in order to prevent backflow of the fluid and minimize tissue damage. Vehicle injections with the same coordinates, volume and speeds were used for sham-lesioned animals.

### 4.3. Behavioral Analyses

Two main types of behavioral parameters were analyzed: sensory–motor performance and spatial learning and memory in the Morris water maze.

#### 4.3.1. Motor Tests

These tests were administered once a week starting from the second week post-lesion and through the subsequent two–three weeks. For each test, the maximum time allowed was 60 s.

In a bridge test, the animals were required to walk across a narrow wooden beam (70 cm long, 3 cm wide) connected to the animals’ home cage. The time spent with all four paws on the beam (equilibrium time) and the latency to cross the beam (walking ability) were recorded.

In a grid test, the animals were placed onto a framed (70 × 70cm) wire mesh screen (3.5 cm/square) connected to the home cage and inclined at 50° from the horizontal plane. The animals were placed in the middle part of the screen facing downward. The latency before the rat turned to face upward and walk towards the home cage, as well as the number of falls into the grids (both providing an index of motor coordination), were recorded.

#### 4.3.2. Morris Water Maze

The Morris water maze (MWM) is a widely used testing paradigm, specifically designed to evaluate spatial learning and memory in rodents as their ability to escape water by the skillful use of environmental cues [51]. The apparatus consisted of a cylindrical tank, 150 cm in diameter, 50 cm deep, filled up to 35 cm with room temperature water (20–22 °C). The tank was located in a small testing room containing many external cues which were visible from within the pool and could be used for orientation. Equally spaced points arbitrarily indicated as North, South, East and West served as start positions and divided the tank into four quadrants. A circular escape platform (10 cm in diameter) was anchored to the bottom of the tank with its top about 2 cm below the water surface, onto which the animal could climb to escape. Four annuli were defined as a circular area in the middle of each quadrant, corresponding to the site where the platform would have been if placed in that quadrant. Starting from about four weeks post-lesion, the animals were first habituated to the pool environment by letting them swim freely for 60 s, then training in the water maze task commenced. First, a three-day cued test was administered, during which the platform surface was clearly visible above water and its position changed randomly on each of four daily trials. Subsequently, two different tests, designed to evaluate reference and working memory abilities, were administered in sequence.

In the reference memory version of the water maze task, the rats were given four trials a day over 5 consecutive days. For each trial, the animal was pseudorandomly released from one of the start locations and given 60 s to find the submerged platform (always placed in the South-West, SW, quadrant) and climb onto it. The rat was allowed to rest for 30 s on the platform and was then placed in the next predetermined starting point. If the animal failed to find the platform in the given time, it was manually guided and left on the platform for 30 s prior to starting the next trial. The latency to find the hidden platform, the distance swum and the swim speed were recorded.

After the last trial of the last testing day, the platform was removed, and a further spatial probe trial was administered. The rat was placed at one of the start locations and allowed to swim freely for 60 s, during which the swim path was plotted, and the number of crossings over the annulus in each quadrant was recorded.

#### 4.3.3. Radial Arm Water Maze

Spatial working memory (i.e., the ability to transient store and rapidly recall/process spatial information relevant for goal-directed behavior) was evaluated using a radial arm water maze (RAWM) paradigm modified from [52,53]. The apparatus consisted of the same pool as above, where six swim alleys (50 cm long, 20 cm wide) and an open central area (roughly 50 cm in diameter) were created. The escape platform, located at the end of one of the arms, was kept in the same position over the four trials of the same day, and it was pseudorandomly moved to a new arm on each of five consecutive days. During each trial, the animal was released from one of the unbaited arms and given 60 s to locate the platform, followed by 30 s intertrial time. If the animal did not find the hidden platform, it was manually guided to and kept on it for 30 s. Entering an arm that has already been visited or did not contain the platform was considered an entry error. For each trial, the latency to locate the platform, as well as the number of arm selection errors made prior to entering the correct goal arm, were recorded. With such design, the most significant improvements in performance took place between the first and the second trial. Therefore, in order to obtain a further measure of working memory, the data were also analyzed in the form of savings, defined as the difference between latency and error scores on trials 1 and 2, expressed as a percentage of the respective scores recorded during trial 1.

### 4.4. Histology

Upon completion of behavioral testing, at about 8 weeks post-lesion, the animals under terminal anesthesia (sodium pentobarbital, 60 mg/kg i.p.) were perfused via the ascending aorta with 50–100 mL of room temperature saline followed by 200–300 mL ice-cold phosphate-buffered 4% paraformaldehyde (pH 7.4). The brains were removed, postfixed in the same medium for 2 h and then kept at 4 °C in a phosphate-buffered 20% sucrose solution until they had sunk. Forty µm-thick coronal sections were then cut on a freezing microtome from the prefrontal cortex level through the septal/diagonal band and NBM regions to the level of the caudal hippocampus and collected into four series. Additional series of sections were also cut at the cerebellar level.

One series of sections was processed free-floating for acetylcholinesterase (AChE) histochemistry [54]. Briefly, the sections were incubated at low pH (6.0) in a mixture containing sodium citrate, copper sulfate, sodium acetate, potassium ferricyanide and acetylthiocholine iodide. Ethopropazine (100 µM, Sigma, St. Louis, MO, USA) was added to the incubation medium to inhibit non-specific esterases. The brown reaction product was intensified by brief incubations in ammonium sulfide and silver nitrate. The sections were then mounted and analyzed microscopically.

Alternate sections comprising the basal forebrain nuclei or the cerebellum were processed free-floating for either ChAT or calbindin immunohistochemistry following a standard ABC avidin-biotin protocol. Endogenous peroxidase activity was first quenched by a 10 min incubation in 3% H_2_O_2_ and 10% methanol in 0.02 M potassium phosphate-buffered saline (KPBS, pH 7.4). The sections were preincubated in 5% normal goat serum (NGS, Sigma) and 0.3% Triton X-100 (Merck, Darmstadt, Germany) in KPBS for one hour and then exposed for 16–20 h to rabbit antibodies raised against choline acetyltransferase (ChAT, 1:500; Chemicon, Temecula, CA, USA) or the calcium binding protein calbindin-D_28K_ (1:5000; Swant, Switzerland) diluted in 2% NGS and 0.3 Triton. After a 3 h incubation in a 1:200 goat antirabbit biotinylated IgG (Vector, Burlingame, CA, USA), the sections were incubated in avidin-biotin complex (1 h) and then reacted with 0.025% diaminobenzidine (Sigma) and 0.01% hydrogen peroxide in KPBS for 2–5 min. Sections were mounted, dehydrated, coverslipped and analyzed microscopically. Tissue processing and staining were carried out under identical conditions, using all relevant sections simultaneously so as to ensure consistency during the subsequent morphometric assessments. In order to check for non-specific labeling, some sections were processed omitting the primary antibody. All staining procedures were performed at room temperature.

### 4.5. Microscopical Analyses and Quantitative Estimations

All analyses were carried out on coded slides and the observers were blind to groups’ identity. The extent of lesion-induced cholinergic cell loss in the septum/diagonal band and NBM was estimated stereologically using the optical fractionator principle [55]. Analyses on ChAT-stained sections were conducted using selection criteria as previously described [56]. Immunoreactive neurons in the septum/vDBB were counted bilaterally from the genu of the corpus callosum, rostrally, to the crossing of the anterior commissure, caudally, defining the lateral edge of vDB at the medial border of the olfactory tubercle. Immunoreactive neurons in the NBM were bilaterally counted from the level of the caudal septum to the tail of the caudate-putamen.

Purkinje cell loss was estimated in coronal sections from the anterior cerebellum (defined as containing the simple lobule, planes −10.4/−11.4 according to [50]). Calbindin-immunoreactive Purkinje cells, easily identifiable also by their typical arrangement, were counted in layers, starting from the dorsal surface, in a counting field comprising the dorsal lobules of the vermis (lobules V–VI), dorso-ventrally, and the simple lobe medio-laterally, setting the lateral border at the parafloccular sulcus. About ten alternate sections were sampled from each animal, and only layers which extended straight across the counting field were included in the estimation. The sampling system was an Olympus CAST-Grid System (Olympus Denmark A/S, Albertslund, Denmark). The CAST-Grid software (version 2.0) was used to delineate the selected areas at 4× magnification, and to generate counting frames which were moved randomly and systematically until the entire delineated area had been sampled. Using a 100× oil objective, unambiguously immunoreactive cells were identified and counted after excluding guard volumes from both section surfaces, in order to prevent problems of lost caps.

Relative levels of cholinergic innervation density in the neocortex and hippocampus were measured in AChE-stained sections from intact, vehicle-treated and 192 IgG-saporin-injected animals by densitometry using the Image 1.61 NIH freeware [57], as previously described [37]. Five separate measuring fields of consistent size (0.5 mm in diameter) were selected bilaterally from three different sections in the frontal and parietal cortices, as well as in the dorsal hippocampus. Background staining, as measured in a structure normally lacking the AChE reaction product (i.e., the corpus callosum), was subtracted from each measurement. Relative levels of optical density in the analyzed regions were expressed as arbitrary units, averaging the values obtained from both sides.

### 4.6. Statistical Analyses

The data were seen to comply with the criteria for normal distribution, and they were therefore analyzed using parametric tests for all statistical comparisons. Group differences in behavioral performance, neuron numbers and innervation density were assessed using either one-way analysis of variance (ANOVA) or two-way mixed ANOVA, as appropriate, followed by the Tukey HSD post hoc test. Data are presented as means ± standard error of the mean (sem) and differences are considered significant at *p* < 0.05.

## 5. Conclusions

Evidence accumulating from clinical and neuroimaging studies have supported the view that the cerebellum, besides its classical role in motor control, is largely involved in emotion, language and cognition [49,58,59,60,61]. Surprisingly, however, the cerebellar contribution to major neurodegenerative diseases such as AD has, over the years, received rather scant attention, possibly because ataxia, a motor disturbance typically associated with cerebellar degeneration, as well as amyloid or tau pathologies in the cerebellar tissue have rarely been described in AD patients (e.g., [13,62,63,64,65]). Notwithstanding, the cerebellum has increasingly been recognized to undergo anatomical and metabolic alterations, as well as profound changes in functional connectivity with cortical regions, during AD progression [66].

Loss of Purkinje cells (i.e., the sole output of cerebellar cortex) in AD is a rather debated issue, with some studies reporting significant reductions, particularly in the vermis and the anterior lobe (e.g., [13,16]), whereas other studies do not [66,67].

Clearly, the possibility to ablate discrete populations of Purkinje cells, particularly those expressing the p75NTR that can be selectively targeted by the 192 IgG-saporin immunotoxin, as performed in the present study, offers a novel powerful paradigm to investigate the role played by these cells. Moreover, an important notion substantiating the cerebellar contribution to the AD cognitive profile is that the cortico-hippocampal and cerebellar neuronal populations are part of an interconnected network [68] and may thus degenerate and die together [14,16,49]. In such a framework, the multiple toxin injection design adopted here provides a unique opportunity to study the possible interplay between focal cerebellar degeneration and the selective loss of transmitter-specific regulatory neuron systems (e.g., those in the cholinergic basal forebrain) acting on cortical and hippocampal neurons involved in cognitive processing. Further analyses of cerebellar and basal forebrain alterations in memory dysfunction will therefore be necessary to better understand the nonmotor functions of the cerebellum and its involvement in disease-related dementia.

## Figures and Tables

**Table 1:** Motor performance.

	Equilibrium Time on Ramp (%)	Latency to Cross Ramp (s)	Latency to Reverse on Grids (s)	Number of Falls in Grids
Control (12)	97.8 ± 10.8	7.0 ± 0.4	6.6 ± 0.6	2.4 ± 0.6
BF (12)	99.0 ± 9.9	7.2 ± 0.3	5.9 ± 0.6	3.3 ± 0.4
CBL (12)	98.6 ± 7.2	6.9 ± 0.4	6.2 ± 0.7	2.4 ± 0.5
BF-CBL (12)	97.4 ± 7.5	7.1 ± 0.4	6.3 ± 0.8	2.6 ± 0.6
ICV (12)	97.7 ± 7.1	6.7 ± 0.4	6.3 ± 0.7	2.9 ± 0.4

Simple motor tests were administered on all animals starting from about 2 weeks post-lesion, and consisted in the evaluation of postural and locomotive form onto a 80 cm-long wooden ramp (maintained either horizontal or with a 45° inclination) or an inclined (75°) grid, both connected to the animals’ home cage. Parameters to be analyzed were the % balance time onto the ramp and the time required to cross it, as well as the latency to reverse direction and the number of falls when placed onto a grid. Numbers represent the mean of four determinations ± SEM.

**Table 2:** Morphometric analyses.

	ChAT MS/DBB	ChAT NBM	AChE Fr-ParCx	AChE HPC	CALB Vermis	CALB Hemisph
Control (12)	9552 ± 204	2924 ± 186	97.1 ± 3.7	103.7 ± 5.1	10,117 ± 360	9824 ± 402
BF (12)	254 ± 31 *	179 ± 8 *	25.7 ± 2.5 *	31.6 ± 1.1 *	9532 ± 324	9239 ± 340
CBL (12)	9384 ± 241	2784 ± 110	94.4 ± 1.9	99.5 ± 4.0	3794 ± 285 *	3775 ± 166 *
BF-CBL (12)	367 ± 63 *	174 ± 19 *	25.3 ± 2.2 *	30.6 ± 1.5 *	3416 ± 443 *	3813 ± 125 *
ICV (12)	233 ± 35 *	176 ± 32 *	21.2 ± 2.5 *	24.5 ± 2.1 *	3945 ± 281 *	3671 ± 183 *

Average numbers of ChAT-immunoreactive neurons in the medial septum/diagonal band of broca (MS/DBB) and nucleus basalis magnocellularis (NBM) and of calbindin (CALB)-immunoreactive neurons in the cerebellar vermis and hemispheres (pooled) were estimated by stereology. The density of the AChE-positive innervation in the fronto-parietal cortex (Fr-Par Cx) and hippocampus (HPC, pooling together readings from CA1, CA3 and dentate gyrus) was estimated by densitometry, and it is expressed as arbitrary units. Asterisks indicate significant difference from Control (*p* < 0.01).

## Data Availability

Data are available on request from the C.A.

## Abbreviations

The following abbreviations are used in this manuscript:

ACh	acetylcholine
AChE	acetylcholinesterase
AD	Alzheimer’s disease
BF	basal forebrain
CBL	cerebellum
ChAT	choline acetyltransferase
HBSS	Hank’s balanced salt solution
KPBS	potassium phosphate-buffered saline
MWM	Morris water maze
NBM	nucleus basalis magnocellularis
NGF	nerve growth factor
NGS	normal goat serum
RAWM	radial arm water maze
Sept/vDBB	septum/vertical limb of the diagonal band of broca
SW	south-west
